# *Burkholderia pseudomallei* invades the olfactory nerve and bulb after epithelial injury in mice and causes the formation of multinucleated giant glial cells *in vitro*

**DOI:** 10.1371/journal.pntd.0008017

**Published:** 2020-01-24

**Authors:** Heidi Walkden, Ali Delbaz, Lynn Nazareth, Michael Batzloff, Todd Shelper, Ifor R. Beacham, Anu Chacko, Megha Shah, Kenneth W. Beagley, Johana Tello Velasquez, James A. St John, Jenny A. K. Ekberg

**Affiliations:** 1 Menzies Health Institute Queensland, Griffith University, Southport, Australia; 2 Clem Jones Centre for Neurobiology and Stem Cell Research, Griffith University, Nathan, Australia; 3 Institute for Glycomics, Griffith University, Southport, Australia; 4 Institute for Health and Biomedical Innovation, School of Biomedical Sciences, Queensland University of Technology, Brisbane, Australia; 5 Trinity College Institute of Neurosciences, Trinity College, Dublin, Ireland; 6 Griffith Institute for Drug Discovery, Griffith University, Nathan, Australia; University of Texas Medical Branch, UNITED STATES

## Abstract

The infectious disease melioidosis is caused by the bacterium *Burkholderia pseudomallei*. Melioidosis is characterised by high mortality and morbidity and can involve the central nervous system (CNS). We have previously discovered that *B*. *pseudomallei* can infect the CNS via the olfactory and trigeminal nerves in mice. We have shown that the nerve path is dependent on mouse strain, with outbred mice showing resistance to olfactory nerve infection. Damage to the nasal epithelium by environmental factors is common, and we hypothesised that injury to the olfactory epithelium may increase the vulnerability of the olfactory nerve to microbial insult. We therefore investigated this, using outbred mice that were intranasally inoculated with *B*. *pseudomallei*, with or without methimazole-induced injury to the olfactory neuroepithelium. Methimazole-mediated injury resulted in increased *B*. *pseudomallei* invasion of the olfactory epithelium, and only in pre-injured animals were bacteria found in the olfactory nerve and bulb. *In vitro* assays demonstrated that *B*. *pseudomallei* readily infected glial cells isolated from the olfactory and trigeminal nerves (olfactory ensheathing cells and trigeminal Schwann cells, respectively). Bacteria were degraded by some cells but persisted in other cells, which led to the formation of multinucleated giant cells (MNGCs), with olfactory ensheathing cells less likely to form MNGCs than Schwann cells. Double Cap mutant bacteria, lacking the protein BimA, did not form MNGCs. These data suggest that injuries to the olfactory epithelium expose the primary olfactory nervous system to bacterial invasion, which can then result in CNS infection with potential pathogenic consequences for the glial cells.

## Author summary

Infections of the central nervous system (CNS), though uncommon, are associated with severe morbidity and mortality. *Burkholderia pseudomallei*, the causative agent of melioidosis, can infect the CNS. We have shown that *B*. *pseudomallei* can enter the CNS via peripheral nerves extending between the nasal cavity and the brain (bypassing the blood-brain/blood-cerebrospinal fluid barriers). In the current study, we show that prior injury to the olfactory epithelium can increase *B*. *pseudomallei* invasion of the olfactory nerve and bulb, highlighting a novel risk factor for CNS infections. We also demonstrate the ability of peripheral nerve glia to internalise *B*. *pseudomallei*, resulting in the formation of multinucleated giant cells (MNGCs), dependent on the bacterial protein BimA. These findings provide important new insights into the pathogenesis of *B*. *pseudomallei*.

## Introduction

*Burkholderia pseudomallei*, a facultative gram-negative bacillus commonly found in soil and stagnant water throughout southeast Asia and northern Australia, causes the multisystem disease melioidosis. Infection is considered to occur by percutaneous inoculation or by inhalation, particularly during the rainy season [1]. Symptoms range from skin and nasal infections to systemic presentations with pneumonia and septic shock [2]. Melioidosis causes ~90,000 deaths annually [3]. The fulminating septicaemia form of melioidosis has a mortality rate of ~90% [4]. Melioidosis is considered severely under-reported and *B*. *pseudomallei* could be endemic to half the countries in the world [3]. *B*. *pseudomallei* is predicted to increase in incidence and spread with climate change [5], and has been considered a potential bioweapon [6]. Diabetes mellitus is a major predisposing factor for melioidosis [7] and contracting the disease is a serious threat to immunocompromised people [8]. *B*. *pseudomallei* can cause CNS infections (neurological melioidosis), which are ~five times more common in Australia than southeast Asia (constituting ~5% of Australian melioidosis cases), and are associated with a high mortality rate and serious sequelae ([9–11], reviewed in [12]).

We have previously shown that in mice, the nerves extending between the nasal cavity and the brain constitute paths by which *B*. *pseudomallei* can invade the CNS. These nerves are the olfactory nerve, which extends between the nasal epithelium and olfactory bulb, and the trigeminal nerve, which connects the nasal cavity and the brainstem. Thus, these nerves provide direct conduits between the nasal cavity and the CNS. [13]We have previously shown that *B*. *pseudomallei* rapidly (within 24 h of intranasal inoculation) reached the olfactory bulb via the olfactory nerve, or the brainstem and spinal cord via the trigeminal nerve in mice [14–18]. One study identified thickening of the trigeminal nerve in three out of seven human neurological melioidosis patients, indicative of nerve invasion to the CNS, bypassing the blood-brain barrier. The same three patients were also exhibiting signs of sinusitis [13]. We have also shown that the bacterial protein *Burkholderia* intracellular motility A (BimA), which mimics a eukaryotic actin polymerase to mobilise a tail of host cell actin leading to bacterial motility, cell-cell dissemination and cell-cell fusion, is important for CNS invasion [18]. We have also found that the nerve path to the CNS was dependent on mouse strain. In inbred Balb/C mice, *B*. *pseudomallei* infected both the olfactory and trigeminal nerves [14–17]. In contrast, in our S100β-DsRed mouse line (outbred Quackenbush Swiss strain), only the trigeminal nerve became infected [18], highlighting the difference in immunological responses between mouse strains; such differences have previously been shown between Balb/C mice and other strains [19, 20].

The olfactory nerve (cranial nerve I) is the shortest cranial nerve, extending between the olfactory neuroepithelium and the olfactory bulb in the forebrain. The cell bodies of primary olfactory neurons are found in the neuroepithelium; their dendrites extend into the nasal cavity and their axons together constitute the olfactory nerve, which is unique in that its neurons continuously regenerate [21–23]. Pathogen- or chemical-induced damage to the olfactory epithelium is common and can result in death of olfactory neurons and anosmia. If the injury does not involve damage to the CNS, the anosmia is temporary due to the regenerative capacity of the system [24–29]. However, injury to the olfactory epithelium can lead to removal of the protective mucosal barrier and death of olfactory neurons, resulting in open channels from the olfactory epithelium to the bulb [30, 31]. Thus, to date, it is currently unknown whether it is possible for epithelial injury to result in a transient increased risk of pathogens gaining access to the olfactory nerve and then the CNS. We have found that in Balb/C mice, where *B*. *pseudomallei* can invade the olfactory nerve and bulb, the infection itself caused local direct structural damage to the olfactory epithelium [17]. This resulted in death of primary olfactory neurons immediately underneath the damaged epithelium, leaving empty conduits surrounded by glial cells. It was precisely at these sites of damage that the bacteria were able to penetrate the epithelium and enter the underlying nerve [17]. We therefore hypothesised that injury to the olfactory neuroepithelium increases the risk of *B*. *pseudomallei* invasion of the olfactory nerve and bulb. To test this hypothesis, we investigated whether experimental injury to the olfactory epithelium would allow *B*. *pseudomallei* to penetrate the olfactory neuroepithelium, olfactory nerve and olfactory bulb in our mouse model normally resistant to primary olfactory nervous system invasion (S100β-DsRed Quackenbush Swiss mice). In these mice, intranasal inoculation of *B*. *pseudomallei* resulted in bacterial penetration of the trigeminal nerve, but not the olfactory nerve [18].

We have extensively used the methimazole injury model to investigate mechanisms of olfactory nerve regeneration and glial responses to olfactory nerve injury. Methimazole, a drug used to treat hyperthyroidism, causes tissue-specific death of olfactory neurons secondary to degeneration of the olfactory epithelium in rodents [32]. We have found that methimazole induces patchy epithelial damage interspersed with intact epithelium, which constitutes a more realistic model of olfactory nerve injury than that caused by other neurotoxins or chemical irrigation [33–36]. We therefore used the methimazole model to determine whether epithelial injury increases the risk of *B*. *pseudomallei* invasion of the olfactory nervous system.

The cellular mechanisms in *B*. *pseudomallei* infection of peripheral nerves remain unknown. In the current study, we also investigated how the glial cells populating the olfactory and trigeminal nerves, olfactory ensheathing cells (OECs) and trigeminal Schwann cells (TgSCs), responded to *B*. *pseudomallei*, and a *B*. *pseudomallei* mutant lacking the BimA protein, *in vitro*.

## Materials and methods

### Bacterial strains

The *B*. *pseudomallei* strain MSHR520 is a clinical isolate from a human case of melioidosis, donated by Bart Currie (Menzies School of Health Research, Darwin, Australia). The genome sequence of this strain is available at www.ncbi.nlm.nih.gov/assembly/GCF_000583835.1/.

The current study used an allele replacement mutant of MSHR520 lacking capsule (MSHR520Δ_Cap_). For the *in vitro* experiment assessing the importance of BimA in MNGC formation, a mutant lacking both capsule and BimA (MSHR520Δ_Cap_*Δ*_BimA_) was used; this double mutant strain was derived from MSHR520Δ_Cap_. Both MSHR520Δ_Cap_ and MSHR520Δ_Cap_*Δ*_BimA_ have been previously described [16, 37] and used in our studies on *B*. *pseudomallei* invasion of the CNS via cranial nerves [14–18].

### Animals

We have previously generated the S100ß-DsRed transgenic reporter mice in which the human S100ß promoter drives expression of the DsRed fluorescent protein such that cells that express the S100ß promoter express DsRed in the cytoplasm [38]. In these mice, glial cells including olfactory ensheathing cells (OECs) of the olfactory nerve, and Schwann cells of other peripheral nerves express DsRed protein. Macrophages and chrondrocytes also express DsRed protein, but their distinct morphology and anatomical locations enables easy identification and separation from the glial cells.

### Methimazole treatment and intranasal inoculation

5–10 weeks old S100β-DsRed mice were injected with methimazole (Sigma-Aldrich, 50 mg/kg, 10 mg/ml in phosphate buffered saline, PBS) or vehicle (PBS) using intraperitoneal injection according to our published protocol [33–36]. Three days later, animals were intranasally inoculated with MSHR520Δ_Cap_ or vehicle as described previously [17]. A small amount of frozen stock (-80°C in 20% glycerol; 10–50 μl) was streaked onto LB agar containing streptomycin (100 μg/ml), incubated at 30°C for several days, and a single colony used to inoculate liquid RB broth and grown with shaking for 16 h to stationary phase at 37°C. A portion was used for viable count (CFU) determination on LB agar to ensure that the inoculum used was, consistently, a total of 3x10^5^ cells which were resuspend in PBS and delivered as a 5 μl droplet/nostril. N = 3 mice for the control group (PBS injection + PBS inoculation), 3 for the methimazole alone group (methimazole injection + PBS inoculation), 3 for the *B*. *pseudomallei* alone group (PBS injection + *B*. *pseudomallei* inoculation) and 4 for the methimazole + *B*. *pseudomallei* group (methimazole injection + *B*. *pseudomallei* inoculation).

Animals were housed in individually ventilated hepa-filtered cages (IsoCage N–Biocontainment, Tecniplast) with Aspen wood chip bedding. Animals were provided ad lib food pellets (Standard Rat and Mouse Feed, Speciality Feeds) and water. Environmental conditions within the cages were maintained at a constant temperature (19–23°C) and humidity (40–60%) with a 12-hour light and a 12-hour dark cycle. Following exposure to bacteria, mice were monitored twice daily. No clinical signs indicative of neurological complications was observed in any of the animals during the monitoring period.

### Tissue preparation

Mice were sacrificed 7 days post intransasal inoculation by lethal intraperitoneal injection of sodium pentobarbitone (Lethabarb). Heads were fixed in 4% paraformaldehyde (PFA) in PBS overnight at 4°C, followed by decalcification in 20% ethylenediaminetetraacetic acid (EDTA) for four weeks. Heads were embedded in optimal cutting temperature (OCT) medium (ProSciTech) and frozen. Coronal sections (50 μm) were cut using a cryostat (Leica CM1860).

### Immunohistochemistry

Immunohistochemistry was performed as previously described [33, 36]. Rabbit anti-*B*. *pseudomallei* (1:2,000) was used to label *B*. *pseudomallei*. This antibody was made in-house and raised against the sarkosyl-insoluble fraction enriched for outer membrane proteins (RRID:AB_2736920) [39]. We have previously used this antibody to label *B*. *pseudomallei* bacteria and degradation products in tissue sections [18] and cells [37]. The secondary antibody was donkey anti-rabbit Alexa Fluor 488 (Abcam ab150073; 1:300). Class III Beta tubulin was detected with rabbit anti-beta III Tubulin (Abcam ab18207; 1:200); the secondary antibody was donkey anti-rabbit Alexa Fluor 647 (Thermofisher A31573; 1:400). Antibodies were diluted in 2% bovine serum albumin (BSA) with 0.3% Triton X-100 (TX) in PBS. Cryostat sections were first incubated with 2% BSA/TX/PBS for 60 min at room temperature, followed by overnight incubation with primary antibodies at 4˚C. Sections were washed and incubated with secondary antibodies for 1 h. Cell nuclei were stained with 4’6-diamidino-2-phenylindole (DAPI).

### Image capture and analysis

Images were captured using a Nikon Eclipse Ti2 epifluorescence microscope and an Olympus FV3000 laser scanning confocal microscope. For comparison between different groups, the same image capture settings, laser intensity and focal depths were used. Images were colour balanced uniformly across the field of view using Adobe Photoshop Creative Cloud 2018 (19.1.4) and compiled into panels using Adobe Illustrator Creative Cloud 2018 (22.1). Three-dimensional (3D) reconstructions were made using Imaris x64 (7.4.2). For detection of whether bacteria were present in the olfactory epithelium, olfactory nerve and olfactory bulb, a minimum of three tissues sections of these areas were analysed per mouse.

For quantification of the number of *B*. *pseudomallei* rods in the olfactory epithelium, rods were defined as anti-*B*. *pseudomallei* immunoreactive rod shapes 1.5–2.5 μm in length. The number of rods was counted in six regions of interest (ROIs; two within the lower epithelium, two within the middle epithelium and two in the upper epithelium) in three tissue sections per mouse (n = 3-4/group). Each ROI area was 440 μm x 440 μm of 50 μm thick tissue sections. Epithelial thickness was measured in three uniform ROIs, in three tissue sections for each mouse (n = 3/group). Statistical analysis was performed using GraphPad Prism 7. Statistical significance between groups assessed using a one-way analysis of variance (ANOVA) with *p*-values of <0.05 considered to represent statistically significant group differences.

### Glial cell culture

OECs and TgSCs were prepared from postnatal day 7 S100ß-DsRed transgenic mice as described previously [15, 40, 41]. Briefly, the olfactory mucosa overlying the nasal septum or the outer layer of olfactory bulb was dissected out for preparations of lamina propria/bulbar OECs, and the trigeminal nerve on the basal surface of the cranial cavity was dissected out for TgSCs. The explants were separately plated in Matrigel (BD Bioscience, 1:10) coated wells in a plastic 24-well plate and maintained in glial medium containing Dulbecco's Modified Eagle Medium with 10% foetal bovine serum (FBS), G5 supplement (Gibco), gentamycin (Gibco, 50 mg/mL) and l-glutamine (200 μM) at 37°C with 5% CO_2_ for 5 days. Cells were replated into plastic 24-well plates and allowed to proliferate to ~80% confluency.

### Cell debris preparation

Fluorescent axon-derived cell debris was generated as described previously [34, 42]. Briefly, the nerve fibre layer of the olfactory bulb of an OMP-ZsGreen mouse [43] was dissected out and partially digested using TrypLE express (Life Technologies) and collagenase (0.1 mg/ml, Life Technologies) for 30 min. After centrifugation to pellet debris, the debris was weighed and resuspended in DMEM to a concentration of 1 mg/ml, triturated using a syringe with a 27-gauge needle and stored at -80°C.

### *In vitro* cell assays

Dilutions of *B*. *pseudomallei* bacteria were prepared in Dulbecco’s phosphate buffered saline (DPBS). OECs and TgSCs were seeded at 5000 cells/well in 8-well chambers (Sarstedt), in glial medium. After 12 h, bacteria (MOI 75:1) and/or cell debris (final concentration: 33 μg/ml) were added and incubated with cells for 24 h. Cells were then rinsed in 1x HBSS and were fixed for 20 min in 4% paraformaldehyde (PFA) in DPBS. Cells were washed and incubated in blocking buffer for 1 h before immunolabelling for *B*. *pseudomallei* as described above. Nuclei were stained with DAPI. Images of the cells were taken with a confocal microscope (Olympus FV1000); the number of MNGCs were counted (n = 5 fields of view with 50–70 cells in each). For the assay which investigated cellular responses to *B*. *pseudomallei* lacking the BimA protein, OECs and TgSCs were incubated with MSHR520Δ_Cap_*Δ*_BimA_ (MOI 75:1) for 48 h.

### Ethics statement

All procedures were approved by Griffith University and the University Animal Ethics Committee (ESK/02/15/AEC) under the guidelines of the National Health and Medical Research Council of Australia and in accordance with the *Australian Code for the Care and Use of Animals for Scientific Purposes (8*^*th*^ *Edition*, *2013*); and in accordance with the Australian Commonwealth Office of the Gene Technology Regulator.

## Results

We have previously shown that intranasal *B*. *pseudomallei* inoculation of S100β-DsRed mice resulted in bacterial penetration of the trigeminal nerve, but not the olfactory nerve, suggesting that the S100β-DsRed mice were normally resistant to *B*. *pseudomallei* invasion of the olfactory nerve [18]. We therefore hypothesised that disruption of the olfactory neuroepithelium may facilitate bacterial invasion and penetration of the olfactory nerve.

### Methimazole treatment, but not *B*. *pseudomallei* infection alone, causes degradation of the olfactory epithelium and olfactory nerve fascicles in S100β-DsRed mice

To determine whether injury to the olfactory nerve altered the ability for *B*. *pseudomallei* to infect the olfactory nerve and bulb in S100β-DsRed mice, we treated mice with methimazole [33, 34], which causes death of primary olfactory neurons [32], and three days later inoculated the mice intranasally with *B*. *pseudomallei*. At this time, death of olfactory neurons is at its peak [33, 34] and methimazole has been cleared, limiting potential side-effects of methimazole [44]. To restrict infection to nerves and not via the haematogenous route, we used a capsule-deficient *B*. *pseudomallei* mutant which cannot survive in the blood (*Δ*_Cap_) [16]. We sacrificed the animals 7 days post infection and analysed tissue sections from the olfactory nervous system for the presence of *B*. *pseudomallei* using immunohistochemistry.

**Fig 1:**
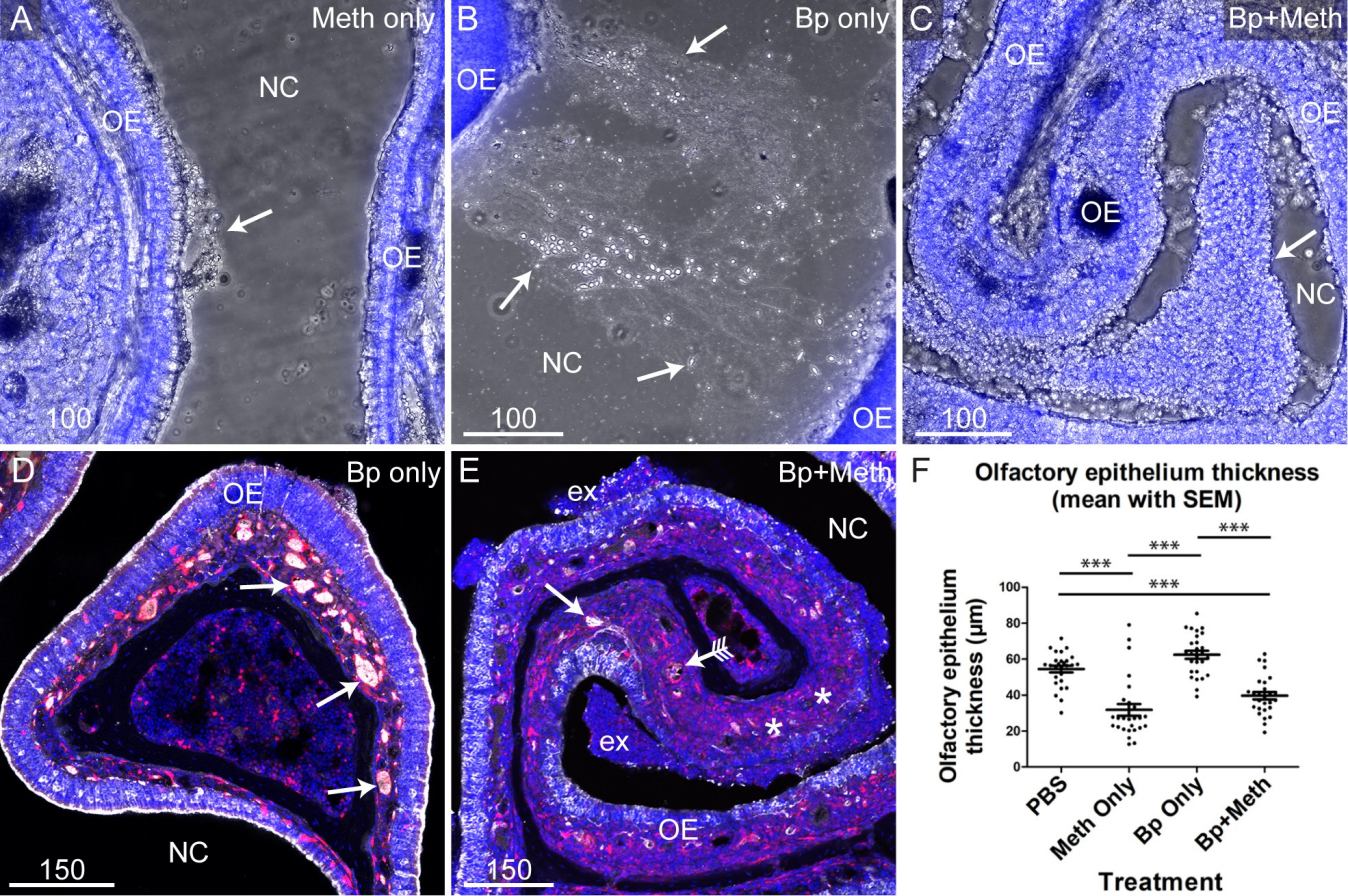
Effect on olfactory epithelium of methimazole pre-treatment and *B*. *pseudomallei* intranasal inoculation. (A-C) Low power images of the nasal cavity (NC) and olfactory epithelium (OE) from mice treated with either methimazole only (Meth only), *B*. *pseudomallei* (Bp only) or methimazole followed by *B*. *pseudomallei* (Bp+Meth). Images show both bright-field (grey) and DAPI (blue, nuclear stain) channels. (A) Arrow points to an area of clustered exudate close to the OE. (B) Arrows are pointing to a large collection of stringy exudate within the NC between areas of OE. (C) Arrow points to an area of clumped exudate close to the OE. (D-E) Panels show sections from S100β-DsRed mice in which OECs express DsRed (red), and immunolabelled for beta-tubulin III (white) with nuclei stained with DAPI (blue). Low power images showing a coronal view of the nasal cavity (NC) in mice treated either with *B*. *pseudomallei* only (D) or methimazole followed by *B*. *pseudomallei* (E). (D) Mice inoculated with *B*. *pseudomallei* only showed very little degradation of the OE with negligible degradation to olfactory nerve fascicles (arrows). (E) Mice pre-treated with methimazole then inoculated with *B*. *pseudomallei* showed OE degradation, exudate (ex) and damaged peripheral nerve fascicles (arrow with tails). There were also areas of degraded OE where no peripheral nerve fascicles were visible (*). OE degradation, while extensive, was not uniform with some peripheral nerve fascicles remaining intact (arrow without tails). (F) Pre-treatment with methimazole (Meth only and Bp+Meth) causes degradation of the olfactory epithelium. Mice treated with vehicle only (PBS) or *B*. *pseudomallei* only did not show extensive olfactory epithelium degradation. 27 data points from three ROIs were measured per mouse (n = 3/group). Graph shows each measured point as a dot with error bar showing the mean plus the standard error of the mean. *** = p <0.001. Scale bars in μm.

**Fig 2:**
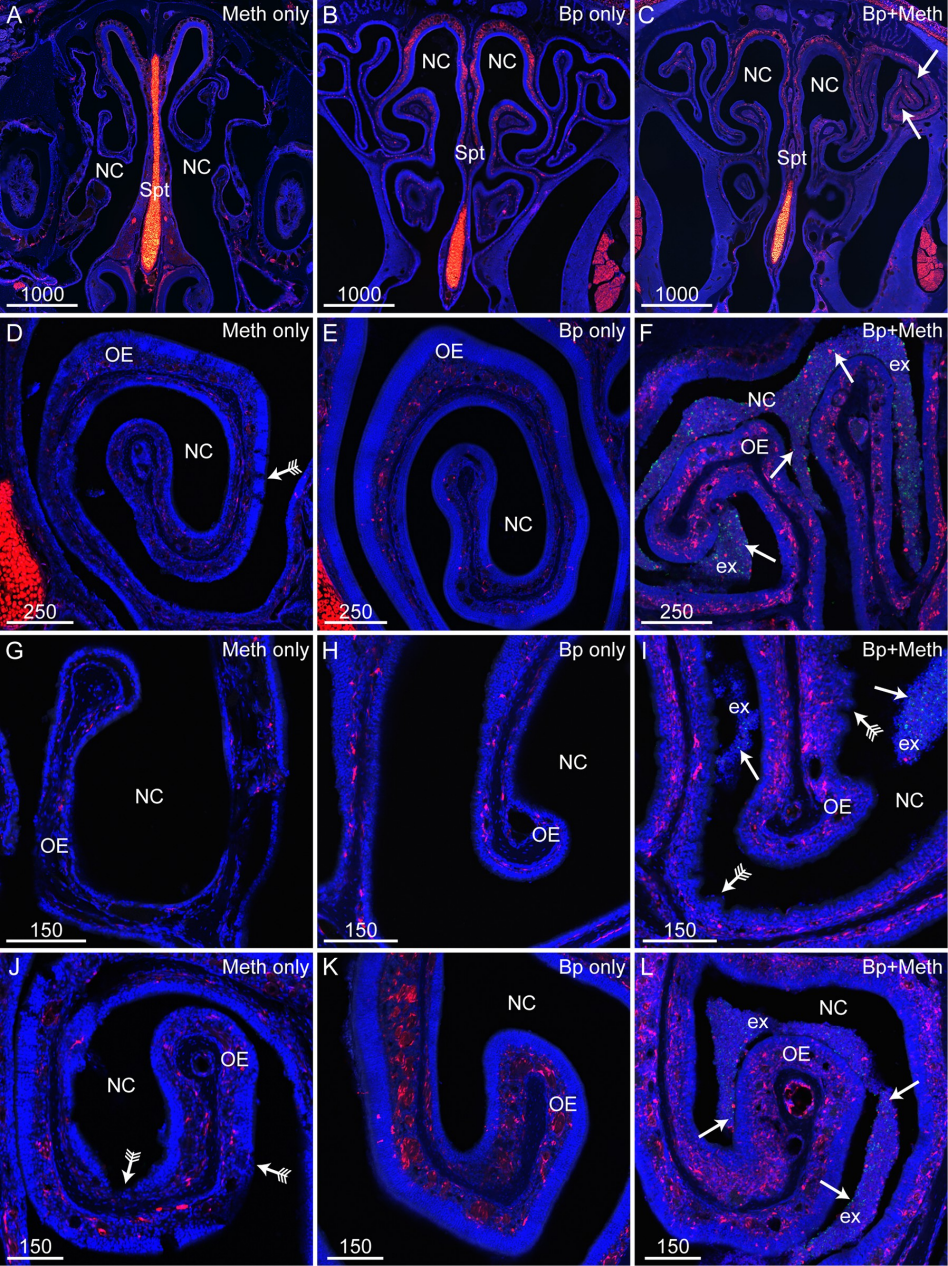
Methimazole pre-treatment causes olfactory epithelium degradation and increased exudate production in mice inoculated with *B*. *pseudomallei*. Panels show sections from S100β-DsRed mice; (OECs and chrondrocytes are red), immunolabelled for *B*. *pseudomallei* (green) with nuclei stained with DAPI (blue). (A-C) Low power images showing a coronal view of the nasal cavity (NC) and septum (Spt) in mice treated either with methimazole only (A), *B*. *pseudomallei* only (B), or both methimazole and *B*. *pseudomallei* (C). (C) Large patches of exudate were present within the nasal cavity (arrows) of mice treated with methimazole prior to intranasal inoculation with *B*. *pseudomallei*. (D-L) Higher magnification showing coronal views of the NC and OE. (D, G, J) Mice treated with methimazole (Meth only) showed patches of OE degradation (arrows with tails). (E, H, K) Mice intranasally inoculated with *B*. *pseudomallei* (Bp only) had intact OE with negligible degradation and exudate present. (F, I, L) Mice first treated with methimazole then intranasally inoculated with *B*. *pseudomallei* (Bp+Meth) showed regions of OE crenellation (arrows with tails) and exudate (ex) containing *B*. *pseudomallei* (green, arrows). Scale bars in μm.

Methimazole treatment caused a drastic change in the appearance of nasal mucous exudate. Mice treated with methimazole alone had small patches of clustered exudate (Fig 1A), whilst mice inoculated with *B*. *pseudomallei* alone typically had stringy exudate (Fig 1B). In contrast, mice pre-treated with methimazole followed by *B*. *pseudomallei* inoculation typically had large clusters of exudate (Fig 1C). We examined the integrity of peripheral nerves fascicles and olfactory epithelium of mice treated with *B*. *pseudomallei* alone, or methimazole followed by *B*. *pseudomallei*. Mice inoculated with *B*. *pseudomallei* alone did not exhibit visible degradation of peripheral nerve fascicles within the olfactory epithelium (Fig 1D). In contrast, patches of peripheral nerve degradation were seen in mice pre-treated with methimazole; however, not all peripheral nerve fascicles were degraded (Fig 1E). We determined the thickness of the olfactory epithelium in the different groups and found that the epithelium was significantly thinner in mice treated with methimazole (alone or followed by *B*. *pseudomallei* inoculation) than in mice not treated with methimazole (treated with either vehicle or *B*. *pseudomallei* alone) (Fig 1F). The data confirms that methimazole causes destruction of the olfactory epithelium and induces death of olfactory neurons and shows that *B*. *pseudomallei* infection alone does not destroy olfactory nerve fascicles in these mice. We further confirmed that methimazole, but not *B*. *pseudomallei* alone, caused epithelial degradation by closely examining the olfactory epithelium (Fig 2). Degradation of the olfactory epithelium was clearly seen in mice pre-treated with methimazole (Fig 2D, 2G and 2J) while no obvious degradation was detected in mice inoculated with *B*. *pseudomallei* only (Fig 2E, 2H and 2K). Mice pre-treated with methimazole and then inoculated with *B*. *pseudomallei* had areas of epithelial degradation, large clusters of mucous exudate containing *B*. *pseudomallei* bacteria (Fig 2F and 2L) and patches where the olfactory epithelium was crenelated (Fig 2I).

### Pre-treatment with methimazole exacerbates *B*. *pseudomallei* infection of the olfactory epithelium

**Fig 3:**
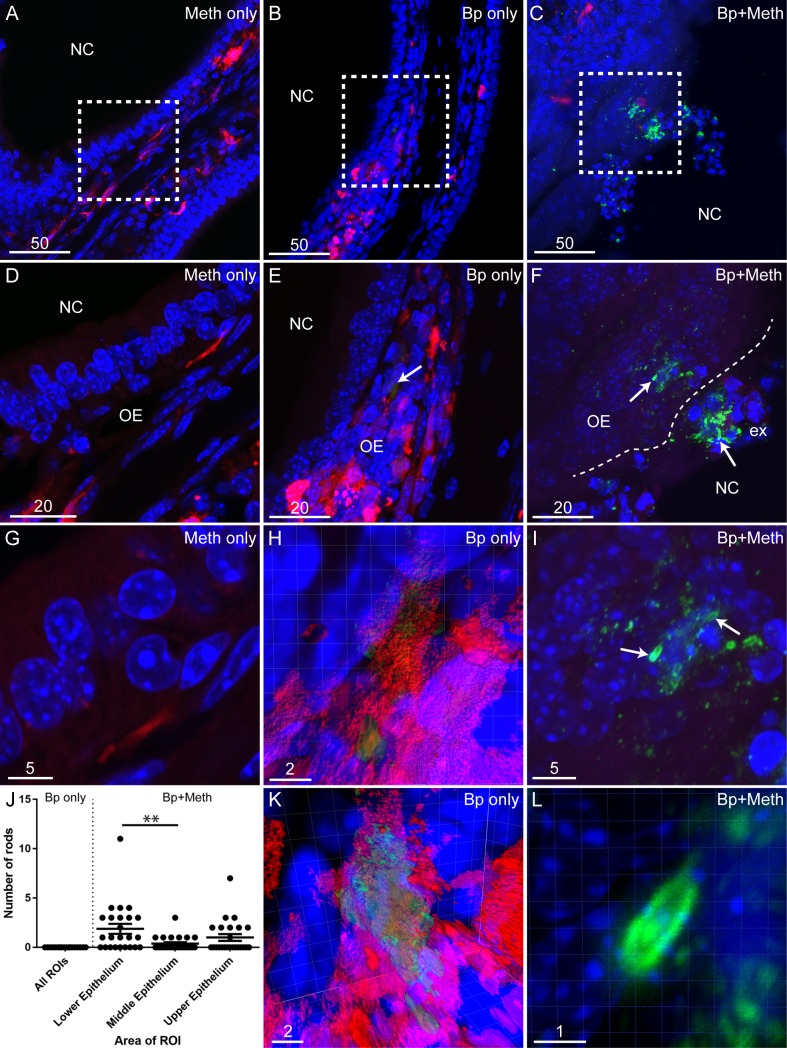
Methimazole treatment exacerbates *B*. *pseudomallei* infection of the olfactory epithelium. Panels show images of coronal sections of the olfactory mucosa. (A-C) Low power images of the nasal cavity (NC) from S100β-DsRed mice, showing OECs (red) and immunolabellingfor *B*. *pseudomallei* (green) with nuclei stained with DAPI (blue). (D) A higher magnification of square in panel A showing the NC and olfactory epithelium (OE) with no immunolabelling seen for *B*. *pseudomallei*. (E) Magnified area of square in panel B. *B*. *pseudomallei* immunolabelling (green) is seen within the OE (arrow), shown at higher magnification below. (F) A higher magnification of boxed region in panel C. Extensive immunoreactivity for *B*. *pseudomallei* (green) is seen in the NC within exudate (ex) and the OE. Arrows show *B*. *pseudomallei* rods within areas of associated particles immunoreactive for anti-*B*. *pseudomallei* antibodies. (G) A very high magnification of the OE showing no immunoreactivity for *B*. *pseudomallei* in mice treated with methimazole alone. (H) A very high magnification and three-dimensional (3D) reconstruction of the *B*. *pseudomallei* immunoreactivity seen in panel E (arrow). (I) A very high magnification of *B*. *pseudomallei* rods (arrows) within the olfactory epithelium (OE) seen in panel F. Associated particles immunoreactive for anti-*B*. *pseudomallei* antibodies can also be seen within the OE. (J) Graph showing the numbers of *B*. *pseudomallei* (Bp) rods within the lower, middle and upper olfactory epithelium of mice pre-treated with methimazole prior to *B*. *pseudomallei* infection (n = 4) with error bars showing the mean plus the standard error of the mean. Within sections of the olfactory epithelium, six ROIs (440 μm by 440 μm in size with 50 μm depth) were defined (two ROIs each for the lower, middle and upper epithelium). For each mouse, three sections containing these ROIs were analysed. There were significantly more *B*. *pseudomallei* rods within the lower epithelium than in the middle epithelium (** = p ≤ 0.01). (K) A rotation of the 3D reconstruction seen in panel H. *B*. *pseudomallei* reactivity (green) appears to be localised within an S100β-DsRed positive cell. (L) A very high magnification and 3D reconstruction of the *B*. *pseudomallei* rod (green) shown in panel I. Scale bars in μm.

Closer examination of the olfactory epithelium further showed clear differences between mice that were only inoculated with *B*. *pseudomallei* and those that were pre-treated with methimazole before inoculation with *B*. *pseudomallei* (Fig 3). Areas containing degraded *B*. *pseudomallei*-derived material (which is also detected by the anti-*B*. *pseudomallei* antibody [17]) were seen in the olfactory epithelium of mice inoculated with *B*. *pseudomallei* only (Fig 3E). Higher magnification imaging showed that the degraded bacteria in mice treated with *B*. *pseudomallei* alone appeared to be localised within DsRed-expressing glia of the olfactory nerve (Fig 3H and 3K). We did not find any intact *B*. *pseudomallei* rods in the epithelium of any of the mice which had not also been treated with methimazole. In contrast, distinct whole *B*. *pseudomallei* rods were frequently found in the epithelium of all mice that had been pre-treated with methimazole before intranasal inoculation with *B*. *pseudomallei* (Fig 3F). These rods were often surrounded by immunoreactive particles which may be *B*. *pseudomallei* degradation products (Fig 3I and 3L) [18]. To gain further insight into how *B*. *pseudomallei* gains access to olfactory nerve fascicles, we also determined which epithelial area was most susceptible to *B*. *pseudomallei* infection after methimazole treatment, we quantified the number of whole *B*. *pseudomallei* rods in representative epithelial areas (lower, middle and upper epithelium). We found that there were significantly more bacteria in the lower epithelium than in the middle or upper epithelium (Fig 3J).

### *B*. *pseudomallei* invades the olfactory nerve and bulb only after methimazole pre-treatment

**Fig 4:**
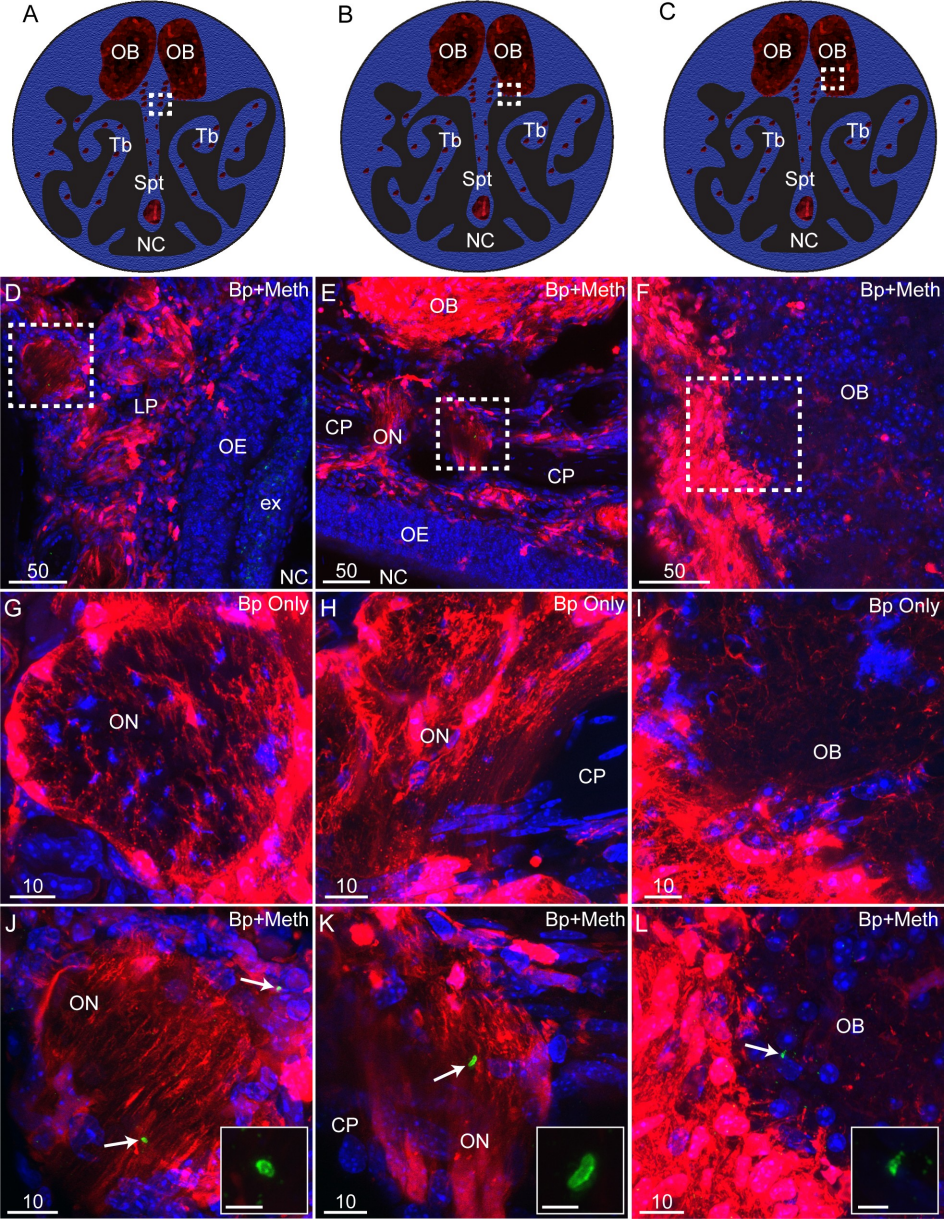
Methimazole pre-treatment causes *B*. *pseudomallei* infection of the olfactory bulb via the olfactory nerve. (A-C) Schematic drawings of a coronally sectioned mouse head showing the nasal cavity (NC), nasal septum (Spt) and turbinates (Tb). Red dots represent peripheral nerve fascicles (olfactory and trigeminal nerves fascicles); the olfactory bulbs are also shown in red (OB). (D-L) All panels show coronal sections from S100β-DsRed mice; D-F were treated with methimazole followed by *B*. *pseudomallei* inoculation (Bp+Meth), while G-I were inoculated *B*. *pseudomallei* only (no methimazole). Sections show OECs (red), immunolabelling for *B*. *pseudomallei* (green), with nuclei stained with DAPI (blue). (D) Location of panel D is shown by the white box in panel A. This is a low power view of the NC showing the olfactory epithelium (OE), exudate (ex), lamina propria (LP) and olfactory nerve (within white box in panel D). (E) Location of panel E is shown by the white box in panel B. Dorsal region of the NC showing the olfactory nerve (ON) passing through the cribriform plate (CP) connecting the OE and the olfactory bulb (OB). (F) Location of panel F is shown by the white box in panel C. This is a low power coronal view of the OB. (G) Location of panel G represented by the white box in panel A. Zoomed image of the ON from a mouse inoculated with *B*. *pseudomallei* only. No *B*. *pseudomallei* was detected within the ON. (H) Location of panel H represented by the white box in panel B. Magnified view of the ON and CP from a mouse inoculated with *B*. *pseudomallei* only. No *B*. *pseudomallei* was detected within the ON. (I) Location of panel I represented by the white box in panel C. Magnified view of the OB from a mouse inoculated with *B*. *pseudomallei* only. No *B*. *pseudomallei* was found within the OB. (J) A zoomed image of the ON shown within the white box in panel D. Arrows point to *B*. *pseudomallei* bacteria (green) present within the ON. (K) A zoomed image of the ON shown within the white box of panel E with an arrow pointing to a *B*. *pseudomallei* (green) rod. (L) A zoomed image of the white box in panel F showing the outer layer of the OB. The arrow indicates *B*. *pseudomallei* (green) present within the OB. (J-L) Smaller images within each panel show a very high magnification of *B*. *pseudomallei* with the scale bar representing 2.5 μm. Scale bars in μm.

**Fig 5:**
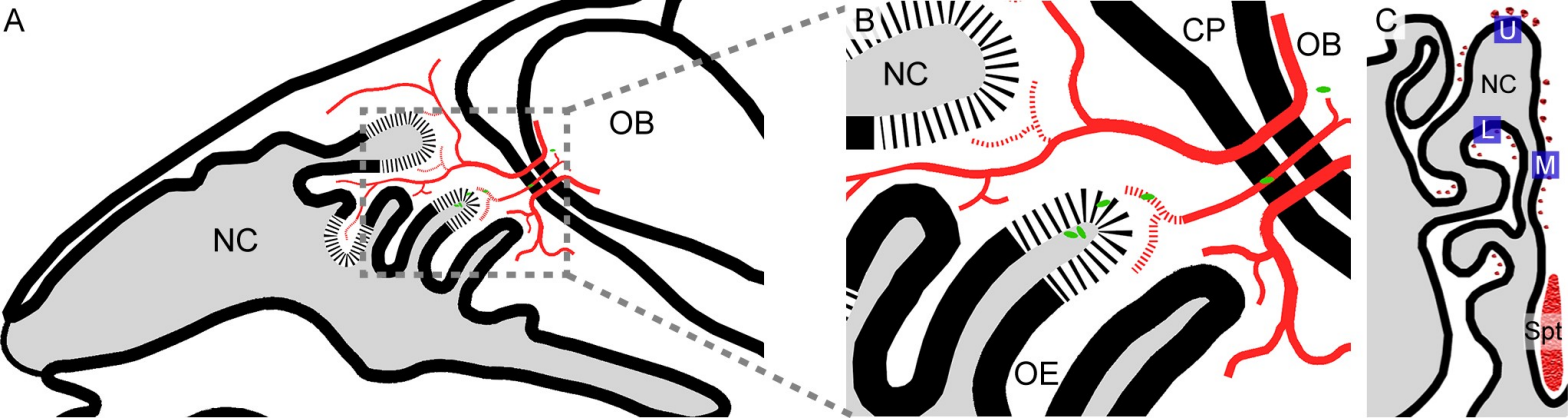
Schematic drawings summarising *B*. *pseudomallei* invasion of the olfactory bulb in mice pre-treated with methimazole. (A-B) A schematic drawing of a sagittal mouse head section showing the nasal cavity (NC), olfactory epithelium (OE), cribriform plate (CP), olfactory bulb (OB) and the olfactory nerve (red). (A) Low power view showing the NC and location of the olfactory nerve (red). The olfactory nerve projects from the OB into the OE. (B) A magnified view of the boxed region in panel A. *B*. *pseudomallei* rods (green) are shown within the nasal cavity (NC) close to degraded olfactory epithelium (OE; degradation depicted as segmented lines). The olfactory nerve (red) projects from the olfactory bulb (OB) into the olfactory epithelium (OE) via the cribriform plate (CP). *B*. *pseudomallei* (green) is shown to invade the degraded olfactory nerve (red; degradation depicted as segmented lines) and penetrate the olfactory bulb (OB). (C) A schematic drawing of a coronal mouse head showing the nasal cavity (NC) and nasal septum (Spt). Red dots represent peripheral nerve fascicles (olfactory and trigeminal nerves fascicles). Blue squares indicate representative anatomical locations for the regions of interest (ROIs) used for *B*. *pseudomallei* rod quantification; lower epithelium (L), middle epithelium (M) and upper epithelium (U).

We next determined whether methimazole pre-treatment allowed *B*. *pseudomallei* to invade the olfactory nerve and bulb, which has not previously been described in this mouse strain [17, 18]. Olfactory nerve axons extend from the olfactory epithelium through the lamina propria, where they fasciculate and project via the cribriform plate to the olfactory bulb, where the axons synapse with their targets (Fig 4A–4C, Fig 5). We only found evidence of *B*. *pseudomallei* bacteria in the pre-treated methimazole group, suggesting the importance of mechanical injury to nerve invasion. In this group, bacteria were found in the olfactory nerve of 75% of mice (Fig 4D, 4E, 4J and 4K). In contrast, no *B*. *pseudomallei* bacteria were found in the olfactory nerve of any of the mice inoculated with *B*. *pseudomallei* alone (Fig 4G and 4H). In mice pre-treated with methimazole, *B*. *pseudomallei* had also invaded the olfactory bulb (also in 75% of the mice; the same mice which showed olfactory nerve infection; Fig 4F and 4L). However, in the olfactory bulb, we detected primarily *B*. *pseudomallei* immunoreactive particles (likely degradation products) (Fig 4J, 4K and 4L). We found no evidence of *B*. *pseudomallei* bacteria or degradation products in the olfactory bulb of mice not pre-treated with methimazole prior to inoculation (Fig 4I). Overall, our findings suggest that injury to the nasal epithelium facilitates invasion of the olfactory nerve and bulb by *B*. *pseudomallei* (summarizing schematic shown in Fig 5).

### *B*. *pseudomallei* can infect olfactory ensheathing cells and trigeminal Schwann cells, leading to formation of multinucleated giant cells

**Fig 6:**
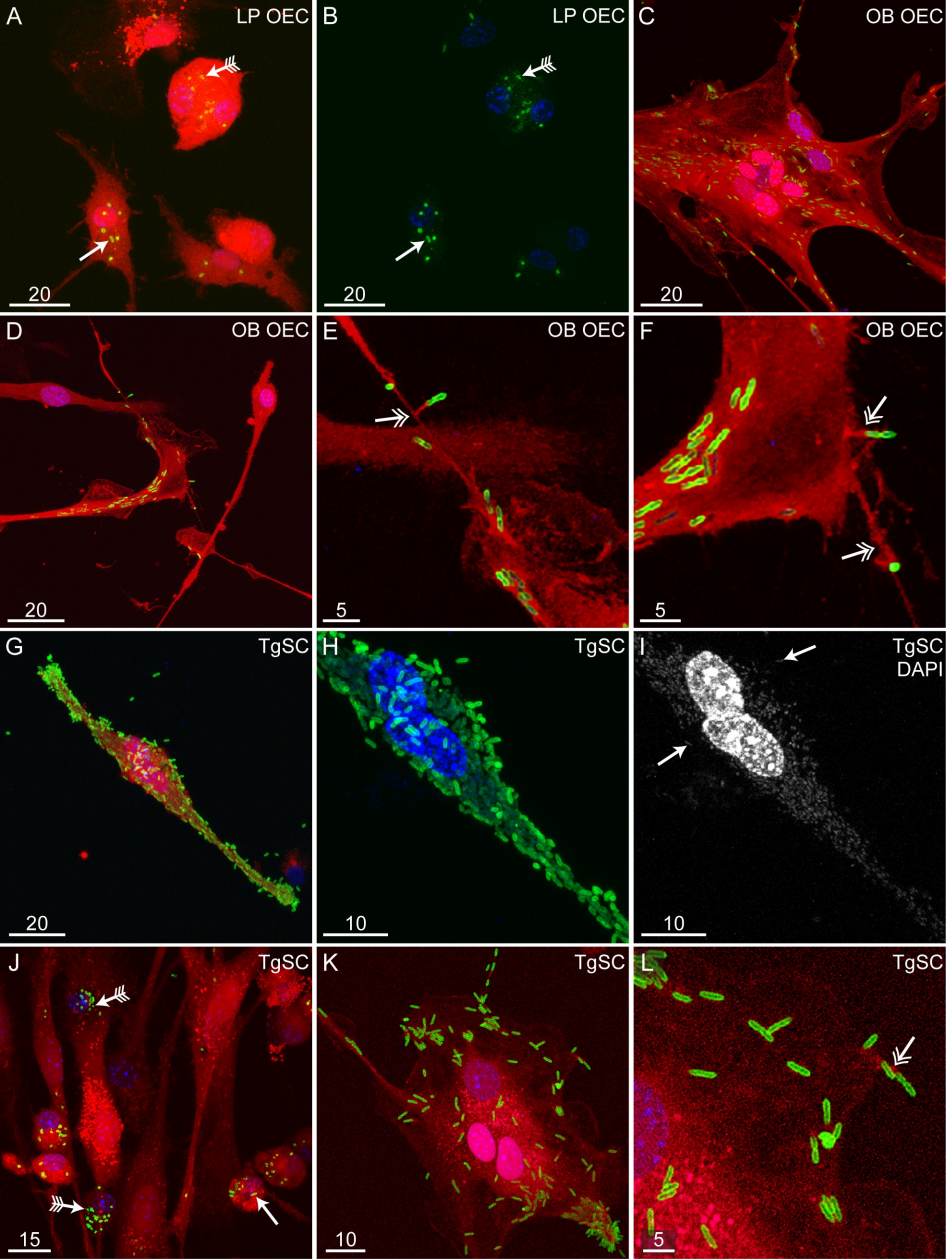
*B*. *pseudomallei* can infect OECs and TgSCs, causing the formation of multinucleated cells. Panels A-B show OECs (red) isolated from olfactory nerve fascicles within the lamina propria (LP), panels C-F show OECs (red) isolated from the olfactory bulb (OB) and panels G-L show TgSCs (red) infected by *B*. *pseudomallei* (MOI 75:1). Cells were infected for 24 h. Nuclei are stained with DAPI (blue) and *B*. *pseudomallei* immunolabelling is shown in green. (A) LP-OECs (red) infected by *B*. *pseudomallei* (green). Whole *B*. *pseudomallei* rods (green; arrow) and degraded bacteria (green; arrow with tails) can be seen. (B) The same image as shown in panel A without the red fluorescence. (C) Multinucleation of OB-OECs (red) after infection with *B*. *pseudomallei* (green). (D) OB-OECs (red) infected by *B*. *pseudomallei* (green); bacteria can also be seen attached to filopodia (zoomed images shown in panels E-F). (E-F) Magnified images of panel D showing OB-OECs (red) with filpodia (double-headed arrows) attached to *B*. *pseudomallei* bacteria (green). (G) Multinucleation of TgSCs (red) after infection with *B*. *pseudomallei* (green). (H) Magnified view of the TgSC shown in panel G infected with *B*. *pseudomallei* (green). In this image, only blue (DAPI; cell nuclei) and green (*B*. *pseudomallei*) fluorescence is shown. The cell has three nuclei. (I) The same image as in panel H, here showing staining of nuclei only (DAPI; grey; arrows). In addition to staining the nuclei of TgSCs, DAPI labels DNA within *B*. *pseudomallei* (arrows). (J) The TgSCs shown (red) appears to have degraded some of the *B*. *pseudomallei* bacteria (green). Whole *B*. *pseudomallei* rods (green; arrow) and degraded bacteria (green; arrow with tails) can be seen within cells. (K) Another example image showing multinucleation of TgSCs (red) after *B*. *pseudomallei* (green) infection. The cell has three nuclei. (L) Magnified image of panel K showing a membrane protrusion (double-headed arrow) of the TgSC (red) attached to *B*. *pseudomallei* (green). Scale bars in μm. Shown are representative images from two biological and three technical repeats.

**Fig 7:**
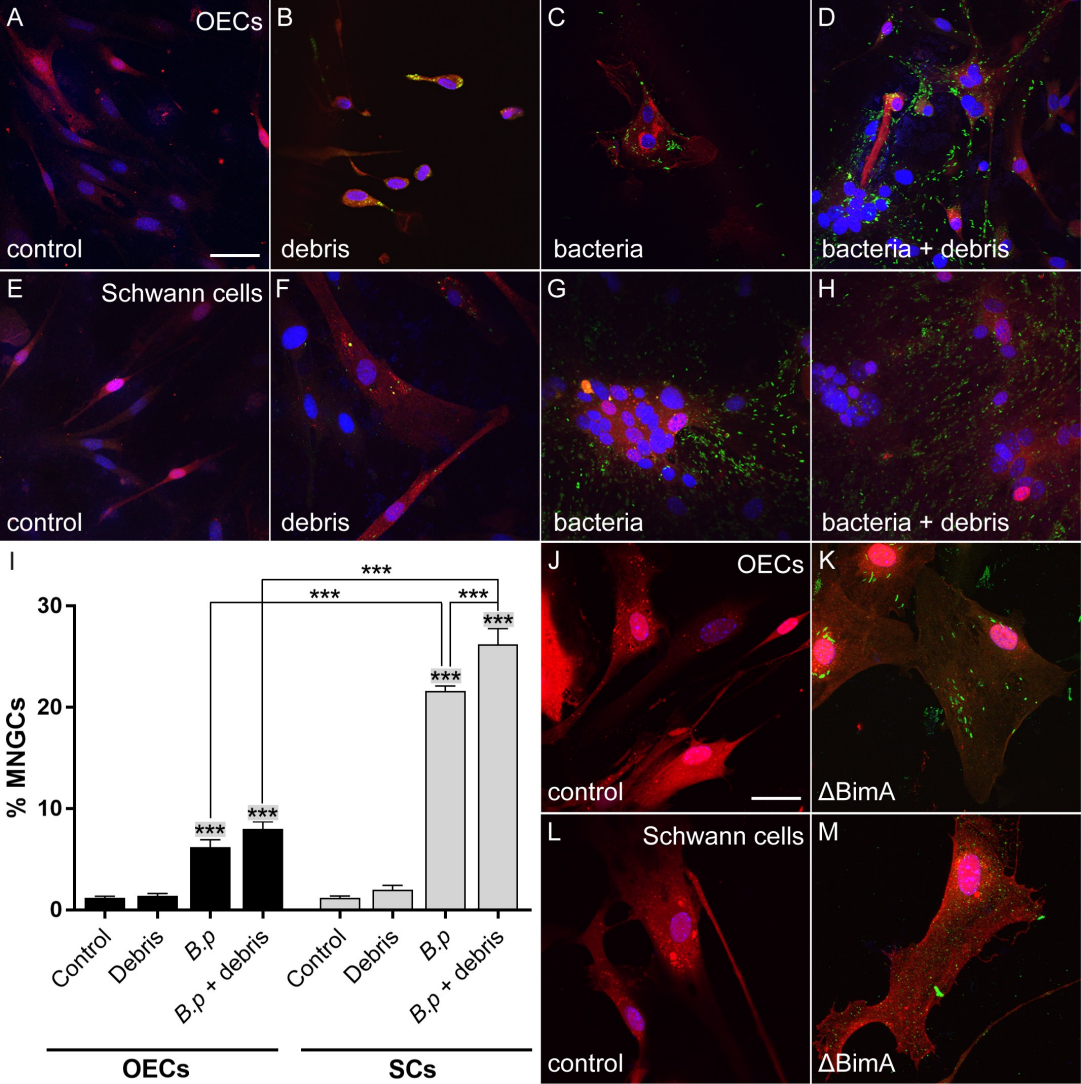
*B*. *pseudomallei*-induced multinucleation of OECs and TgSCs in the absence and presence of axon-derived debris, and example images of glia cultured with *B*. *pseudomallei* lacking BimA. Cells were cultured in the absence/ presence of axonal debris/*B*. pseudomallei for 24 h. Panels A-D show lamina propria-derived OECs (red), panels E-H show TgSCs (SCs; red); nuclei are stained with DAPI (blue). (A) OECs (red) without debris/bacteria (control). (B) OECs cultured with debris derived from ZsGreen-expressing axons (green); the debris was phagocytosed by the cells. (C) OECs cultured with *B*. *pseudomallei* (MOI 75:1) (green). (D) OECs cultured with a combination of *B*. *pseudomallei* and cell debris (both bacteria and debris are green). (E) TgSCs in the absence of debris/bacteria (control). (F) TgSCs with axonal debris. (G) TgSCs with *B*. *pseudomallei*. (H) TgSCs with a combination of *B*. *pseudomallei* and cell debris. Scale bar in A is 15 μm for A-H. (I) Bar graphs show the percentages of multinucleated giant cells in the different conditions (control, debris, *B*. *pseudomallei* (Bp) and Bp + debris) for OECs (black bars) and TgSCs (grey bars). ***significantly different from the control group and from the debris group, p ≤ 0.001. ***significantly different from each other, p ≤ 0.001. N = five fields of view each comprising 50–70 cells (derived from three S100β-DsRed mice); p values are adjusted p values from one-way ANOVA with Tukey’s multiple comparison post-hoc test. (J) OECs (red) cultured without debris/bacteria (control). (K) OECs (red) cultured with *B*. *pseudomallei* ΔBimA (green) for 48 h. (L) TgSCs (red) in the absence of bacteria/debris. (M) TgSCs (red) cultured with *B*. *pseudomallei* ΔBimA (green). Scale bar in J is 15 μm for J-M.

The glial cells of the olfactory and trigeminal nerves are olfactory ensheathing cells (OECs) and trigeminal Schwann cells (TgSCs), respectively. We have shown that these glia are highly phagocytic and can rapidly internalise bacteria [40]. *B*. *pseudomallei* can be internalised into and survive within different types of phagocytes [45–47], which can result in multinucleated giant cell (MNGC) formation as shown in macrophage-like cell lines [37, 48]. To investigate whether *B*. *pseudomallei* could infect peripheral nerve glia and cause the formation of MNGCs, *in vitro* preparations of OECs and TgSCs were cultured with *B*. *pseudomallei*. We found that OECs isolated from olfactory nerve fascicles in the lamina propria and TgSCs from the trigeminal nerve were infected with *B*. *pseudomallei* (Figs 6 and 7), resulting in the formation of MNGCs (Figs 6G–6I, 6K, 7C and 7D, 7G and 7H). In some cells, bacteria were rounded and perhaps degraded; such cells did not form MNGCs (Fig 6A, 6B and 6J). OECs are not only found in the olfactory nerve, but also in the nerve fibre layer of the olfactory bulb, where the olfactory nerve terminates. We also exposed bulbar OECs to *B*. *pseudomallei*; these were also infected, resulting in MNGC formation (Fig 6C–6F). The glia sometimes had membrane protrusions (filopodia) attached to extracellular bacteria (see examples in Figs 6E, 6F, 7L).

### *B*. *pseudomallei* alone, but not axonal debris alone, causes the formation of multinucleated giant cells in olfactory ensheathing cells and Schwann cells

Macrophages can form MNGCs in response to both infection and to injury/presence of cell debris [49–51]. We next investigated whether OECs and TgSCs formed MNGCs in response to cell debris, and/or whether the presence of debris exacerbated the MNGC formation induced by *B*. *pseudomallei*. To mimic a peripheral nerve injury *in vitro*, we challenged OECs (olfactory nerve-derived) and TgSCs with cell debris derived from the olfactory nerve of OMP-ZsGreen mice, in which the olfactory marker protein promoter (OMP) selectively drives expression of ZsGreen in primary olfactory neurons [43]. OECs and TgSCs were exposed to either *B*. *pseudomallei* alone, axonal debris alone or a combination of both *B*. *pseudomallei* and axonal debris (Fig 7). Neither OECs nor TgSCs formed MNGCs after internalising axonal debris in the absence of bacteria (Fig 7B and 7F). In contrast, *B*. *pseudomallei* alone and combined with axonal debris induced the formation of MNGCs in both glial types (Fig 6K, Fig 7C and 7D; 7G and 7H). To compare the extent of MNGC formation between TgSCs and OECs, as well as between the *B*. *pseudomallei* alone and the *B*. *pseudomallei* + debris treatment, percentages of multinucleated cells were calculated for all treatment conditions (Fig 7I). The percentage of MNGCs was significantly higher for TgSCs when compared to OECs for both the *B*. *pseudomallei* alone and the *B*. *pseudomallei* with axonal debris treatment. For TgSCs only, the addition of axonal debris significantly increased the percentage of MNGCs formed compared to inoculation with *B*. *pseudomallei* alone.

To verify that *B*. *pseudomallei* induced MNGC formation due to the action of the BimA protein, as shown for other cell types [37, 52], we then exposed TgSCs and OECs to a mutant *B*. *pseudomallei* strain lacking BimA (Fig 7J–7M). After 48 h incubation, OECs and TgSCs did not form MNGCs (Fig 7K and 7M) and maintained similar morphology to uninfected cells (Fig 7J and 7L), demonstrating that BimA is required for MNGC formation in OECs and TgSCs (Fig 7K and 7M).

## Discussion

Bacterial infections of the brain via the peripheral nerve route are considered rare. The nasal epithelium exhibits a powerful immune defence against pathogens: the nasopharynx-associated lymphoid tissue (NALT). The NALT, which contains lymphocytes and B-/T-cell enriched zones, as well as follicle-associated epithelium with M-cells [53], constitutes the first defence against airborne pathogens [54–56]. Within the underlying olfactory nerve, glial cells (OECs) are efficient phagocytes, capable of engulfing both axonal debris resulting from olfactory nerve turnover and bacteria [34, 40, 42]. Here, we showed that injury to the olfactory epithelium increases *B*. *pseudomallei* infection, allowing bacteria to penetrate the olfactory nerve and bulb in a mouse strain not normally susceptible to *B*. *pseudomallei* infection of the primary olfactory nervous system. These findings may also suggest that outbred mice are more resistant to olfactory nerve infection than inbred mice. Outbred mice are well known to exhibit stronger resistance to infections, to be more “immunocompetent” and to better reflect immune responses in humans than inbred mice [57–65]. Differences in the response to *B*. *pseudomallei* infection has also been demonstrated between different inbred mouse strains. Two studies have shown that after intravenous inoculation, C75Bl/6 mice are significantly more resistant to *B*. *pseudomallei* infection than Balb/C mice [19, 20]. The infection appeared to mimic acute human melioidosis in Balb/C mice, and chronic human melioidosis in C75Bl/6 mice [20]. The difference was attributed to distinct non-specific cellular bactericidal mechanisms [20] as well as different innate and adaptive immune responses [19].

We have previously shown that in inbred Balb/C mice, *B*. *pseudomallei* infection causes significant damage to the otherwise unperturbed olfactory neuroepithelium, allowing invasion of the olfactory nerve and bulb [14–17]. In our outbred S100β-DsRed Quackenbush Swiss mice, *B*. *pseudomallei* epithelial infection and associated damage is minimal, and the olfactory nerve does not become infected [18 and the current study]. We therefore suggest that epithelial damage is central to the ability of *B*. *pseudomallei* to enter the olfactory nerve/bulb. We also showed that there were more bacteria in the lower than in the middle epithelium after methimazole treatment followed by infection, suggesting that the lower epithelium may be particularly sensitive to combined injury/infection. However, the difference was relatively small, and we did not find a significant difference in the number of bacteria between the lower and upper epithelium.

Therefore, is it then possible that injury to the olfactory epithelium can allow other pathogens, including those that do not usually invade the CNS, to infect the brain. One previous study has shown that injury to the nasal epithelium resulted in olfactory nerve and bulb invasion by *Staphylococcus aureus*, which does not invade the unperturbed primary olfactory nervous system [66]. Another study shows that lesions of the olfactory epithelium can accelerate prion invasion of the CNS via the olfactory nerve [67]. These findings open the possibility that epithelial injuries can transiently allow pathogens to enter the CNS via the olfactory nerve route. However, cells within the olfactory bulb may provide a distinct defence against microbial invasion. We have previously shown that in Balb/C mice, where *B*. *pseudomallei* infection leads to patchy epithelial damage and infection of the primary olfactory nervous system, the bacteria are able to bypass the “second defence” barrier of OECs in the olfactory nerve and reach the olfactory bulb (the first defence being the mucosal barrier and NALT) [17]. In the olfactory bulb, however, astrocytes, which form the glia limitans layer, constitute a separate “third defence” barrier and are at least partially capable of degrading the bacteria when they enter the bulb [15]. In the current study, we confirmed that *B*. *pseudomallei* bacteria manage to traverse the olfactory epithelium and can bypass the “second defence” OEC barrier in the olfactory nerve. Within the olfactory bulb, we observed mainly bacterial degradation products and few intact rods (compare Fig 4J and 4K and Fig 4L), suggesting that once *B*. *pseudomallei* infection reaches the bulb, the bacteria become degraded, presumably by the third defence barrier (astrocytes), as we have previously shown in Balb/C mice [15].

Damage to the olfactory epithelium is common, resulting from viruses, bacteria, toxins, traumatic injuries, chemicals and allergies, including smoking [24–29, 68]. Such injuries are typically only noticed if the damage is substantial enough to result in anosmia. Usually, the olfactory epithelium and nerve regenerates relatively rapidly [24–29, 68]. Thus, peripheral injury to the olfactory epithelium and the primary olfactory nervous system is regarded as harmless. The drug used to induce olfactory injury in the current study, methimazole, causes sloughing of sustentacular cells and death of olfactory receptor neurons of the olfactory epithelium in rodents [32, 69, 70] and loss of smell in humans [71–73]. The current study suggests that clinical methimazole treatment [77,78] makes the primary olfactory nervous system vulnerable to bacterial insult. As methimazole is a anti-hyperthroidism drug, it likely has systemic effects which may potentially alter cellular responses within the brain. Thus it may complicate the interpretation of long-term consequences of bacterial invasion on the brain. Alternative nasal epithelial injury models such as mechanical trauma followed by intranasal bacterial inoculation may be more suitable to determine the long-term consequence of bacterial invasion in the brain. It is unknown whether the incidence of CNS infections is higher in patients on methimazole; however, subclinical and latent infections may go unnoticed for long periods of time, as is sometimes is the case for melioidosis [74, 75]. Interestingly, a study from Singapore found that four out of five human patients with neurological melioidosis were also exhibiting signs of sinusitis [76].

Diminished sense of smell, including anosmia, has been identified as a symptom of several neurodegenerative diseases, in particular Parkinson’s disease (PD) and Alzheimer’s disease (AD) [77–84]. The olfactory bulb is the first CNS region to show degeneration in humans with AD and mouse models of familial AD [85–89]; reviewed in [90]. PD is also characterised by abnormalities of the olfactory bulb [91–93]. Studies in mice have shown that a classical hallmark of AD, deposition of amyloid β, occurs first in the peripheral olfactory nervous system before progressing to the olfactory bulb and other CNS areas [94]. One recent study demonstrated that deficits in the olfactory bulb in humans with PD are localised to the ventral bulb, where the olfactory nerve enters [94]. Another study showed that lipopolysaccharide-induced persistent rhinitis led to olfactory bulb damage [95]. Together, these findings support the “olfactory vector hypothesis”–that external agents entering the nasal cavity and damaging the primary olfactory nervous system and olfactory bulb constitute a basis for neurodegeneration [96, 97]. A growing body of work has correlated pathogens with the development of neurodegenerative diseases including AD and PD [98–108]. One known case of post-melioidosis Parkinsonism has been described [109]. The fact that certain pathogens can enter the CNS via the olfactory nerve and bulb [110] suggests that the olfactory vector hypothesis is viable. The current study shows that epithelial injury increases the risk of pathogens invading the CNS via the olfactory nerve, potentially also increasing the risk of pathogen-induced neurodegeneration.

Peripheral glia are thought to play an integral role in the protection of the CNS from bacterial invasion [15, 111]. Thus, it is important to understand the interaction between peripheral glia, such as OECs and TgSCs, and potential pathogens of the CNS. Previous studies have also shown that OECs exhibit a more pronounced response against pathogens than SCs [111]. Our current study showed that OECs and TgSCs can internalise (and become infected by) *B*. *pseudomallei*, with only some cells appearing to degrade the bacteria, suggesting that perhaps there are intrinsic subtypes within the glial populations, exhibiting distinct responses to pathogens. Our images also showed *B*. *pseudomallei* bacteria attached to glial filopodia (Fig 6E, 6F and 6L). It is possible that the cells recognize and attach to the bacteria via these filopodia, similarly to how macrophages detect pathogens [112]. It is also possible that rather than being in the process of being detected by filopodia, the bacteria, via the action of BimA, have mobilised actin tails in the host cell and are in the process of escaping the cell. Indeed, bacterial rods appear attached to the membrane protrusions at the pole (see Fig 6E and 6F), where BimA has previously been shown to be localised [52, 113, 114].

The induction of multinucleated giant cell (MNGC) formation by *B*. *pseudomallei* has previously been described for several phagocytic cell lines [37, 52, 114], although this ability is not correlated with the extent of intracellular replication [37]. Multinucleation occurs via BimA-dependent cell-cell fusion [113]. Surprisingly MNGC formation is not apparent in infected primary monocyte-derived macrophages, or neutrophils [115]. We demonstrate here, for the first time to our knowledge that MNGC formation occurs in phagocytic glial cells. TgSCs were significantly more prone to form MNGCs than OECs. It is possible that this difference suggest that OECs exhibit better or different capacity for phagocytosis of pathogens than TgSCs. OECs constantly phagocytose axonal debris and are naturally exposed to more microorganisms. In contrast, Schwann cells, including TgSCs, only become phagocytic after insult and then also recruit macrophages [reviewed in 116]. The roles of MNGC formation in the pathogenesis of *B*. *pseudomallei* infections are largely unknown but may facilitate localized dissemination and escape from extracellular immune defence [52, 113]. *Mycobacterium leprae*, which survives intracellularly in peripheral nerve Schwann cells, reprograms the host cells towards a de-differentiated phenotype, promoting cell migration and cell-cell dissemination [117]. Because we could not clearly define cell borders in tissue sections, we could not verify that MNGC formation occurred in infected animals. However, if also occurring *in vivo*, MNGC formation may be of significance in the aetiology of neurological melioidosis, considering the role of the trigeminal nerve as a possible route for translocation of *B*. *pseudomallei* to the brainstem [18].

In summary, injury to the nasal epithelium results in increased invasion of the olfactory nerve by *B*. *pseudomallei*. While peripheral glial cells can internalise some of the bacteria, they are susceptible to becoming multinucleated giant cells through a mechanism dependent on the bacterial protein BimA. The ability of *B*. pseudomallei to penetrate nerves of the nasal cavity varies with the strain of mice, suggesting that more extensive studies examining genetic variability may identify the mechanisms by which the bacteria initiate invasion and the associated risk factors. As these results highlight a novel risk factor for CNS infections, future studies should consider the long-term consequences on the low-level presence of bacteria within the brain.
